# The Efficacy of Probiotics for Treatment of Bipolar Disorder-Type 1: A Randomized, Double-Blind, Placebo Controlled Trial

**Published:** 2020-01

**Authors:** Mahin Eslami Shahrbabaki, Saleheh Sabouri, Abdolreza Sabahi, Delaram Barfeh, Parisa Divsalar, Mahdi Esmailzadeh, Atefeh Ahmadi

**Affiliations:** 1 Neurology Research Center, Psychiatry Department of Shahid Beheshti Hospital, Afzalipour Medicine School, Kerman University of Medical Sciences, Kerman, Iran.; 2 Pharmaceutics Research Center, Institute of Neuropharmacology, Kerman University of Medical Sciences, Kerman, Iran.; 3 Neuroscience Research Center and Institute of Neuropharmacology, Psychiatry Department of Shahid Beheshti Hospital, Afzalipour Medicine School, Kerman University of Medical Sciences, Kerman, Iran.; 4 Neurology Research Center, Afzalipour Medicine School, Kerman University of Medical Sciences, Kerman, Iran.; 5 Psychiatry Department of Shahid Beheshti Hospital, Neurology Research Center, Afzalipour Medicine School, Kerman University of Medical Sciences, Kerman, Iran.; 6 Nursing Research Center, School of Nursing and Midwifery, Kerman University of Medical Sciences, Kerman, Iran.

**Keywords:** *Bipolar Disorder*, *Probiotic*, *Placebo*, *Randomized Controlled Trials*

## Abstract

**Objective:** Bipolar disorders are among the most common chronic mental disorders. Despite the recent improvement in controlling psychiatric disorders, treatment of bipolar disorders remains a challenge.

The aim of this study was to determine the effect of consuming probiotics in patients with bipolar disorder-type 1 compared to the placebo group.

**Method** **:** This was a permuted blocked randomized clinical trial conducted in Shahid Beheshti mental hospital in Kerman, Iran, from October 2017 to October 2018. Two psychiatrists diagnosed and hospitalized all 38 patients with type 1 bipolar disorder based on the Diagnostic and Statistical Manual of Mental Disorders (DSM-5). Using blind randomized blocking method size 4, patients were divided into 2 groups of placebo and probiotic. Young Mania Rating Scale (YMRS) and Hamilton's Depression Rating Scale (HDRS) were completed at the beginning, week 4, and week 8 of the study by a psychiatry resident. Independent t test, Mann-Whitney and repeated measures ANOVA tests were used. Data were analyzed using SPSS software version 20.

**Results: **There was no significant difference between the 2 groups in age, sex, and severity of mania and depression. Consumption of probiotics reduced the scores of YMRS and HDRS over time in the probiotic group more than the placebo group, but it was not significant.

**Conclusion: **Consumption of probiotics had non-significant effects on improvement and treatment of bipolar type 1 patients. It is suggested that future studies be conducted with different probiotic microbial strains and longer period of treatment.

The environment of the digestive system is the habitat of several microorganisms that help human health (1). Disproportions of the normal flora of the intestine have been reported in some diseases such as inflammatory bowel disease (IBD), obesity, type 2 diabetes, nonalcoholic steatohepatitis, atherosclerosis, digestive system, and prostate cancers (2, 3, 4). Probiotics prevent the growth of pathogens, trigger the synthesis of some vitamins, preserve the homeostasis of the mucosa, and regulate the body immune system, and they even affect the nervous system. 

Alterations in the normal flora of the intestine have been shown in some of the brain-related disorders, eg, autism spectrum disorders (5, 6), multiple sclerosis (7), and mood disorder. Studies have shown that stress alters the gastrointestinal microbiota balance (8). Some studies investigated the relationship between the effect of probiotic microorganisms on controlling the symptoms and prevention of rehospitalization in mental disorders, including bipolar disorders (20, 21). Probiotic concentration of Faecalibacterium was significantly lower in bipolar patients than in the nonbipolar (9).

Study of Pinto-Sanchez et al in 2017 showed that consumption of probiotic Bifidobacterium longum both reduces depression scores and alters brain activity and moderates response to fear stimuli in various brain regions (10). A similar study on healthy people showed that consumption of probiotic yoghurt for 20 days resulted in mood improvement (12). Inconsistent with previous research, a study by Tomasic et al in 2015 on schizophrenic patients found no significant difference in the severity of psychotic symptoms among schizophrenic patients receiving probiotic supplementation and placebo group; however, immunomodulatory effects from probiotics were noted (11). Emiko Aizawa et al in 2018 found no significant difference between the amount of Bifidobacterium and Lactobacillus bacteria in the stools of bipolar patients and healthy people; however, a negative correlation was found between Lactobacillus levels and people’s sleep. Moreover, there was no significant relationship between the number of fecal bacteria and mania and depression symptoms. This study was conducted on patients with low severity type 1 and 2 bipolar disorder, some of whom used antipsychotics, antidepressants, and mood stabilizers (13). 

Bipolar disorders (BD) are a leading global cause of disability (23) and chronicity (24). Despite the enormous burden of bipolar disorders, the underlying digestive mechanisms associated with disease pathogenesis and progression are still not fully understood. Furthermore, the efficacy of seemingly diverse therapies used in BD appear to share common effects on oxidative, inflammatory, and neurotrophic pathways (25). The potential role of intestinal microbiota in the etiology of various human diseases has attracted considerable attention during the last decade. Considering the possible beneficial effects of probiotics and limited studies on their effects on patients with bipolar disorder worldwide, including Iranian population, in this study, it was aimed to investigate the probiotic consumption effects in patients with bipolar disorder (type 1) compared to placebo.

## Materials and Methods

Study Procedure: This was a double-blind randomized controlled trial using placebo control that was performed at Shahid Beheshti hospital of Kerman Medical University. With census population sampling, all patients who met the inclusion criteria were enrolled in the study from October 2017 to October 2018. However, to estimate the high power of the study, the sample size was calculated based on the below formula: 


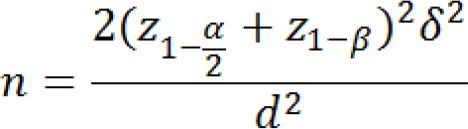


Considering standard deviation (δ) of 6, valuable group difference (d) of 6, type I error (α) of 0.05, type II error (β) of 0.1, and power of 90%, the calculated sample size was 21 patients in each group.

Two psychiatrists diagnosed and hospitalized 38 male and female patients with type 1 bipolar disorder based on the Diagnostic and Statistical Manual of Mental Disorders (DSM-5). 

***Study Sample***

The inclusion criteria in the study, in addition to the diagnosis of type 1 bipolar disorder based on DSM-5 criteria, were age 18-65 years, not consuming any medication or discontinuing it within 2 weeks prior to the study, and not receiving ECT since 4 weeks prior to the study. The exclusion criteria were pregnancy and breast feeding, alcohol and drug use, suicide risk, use of probiotics and supplements over a period of 2 months before the start of the study, chronic diseases (cardiovascular, kidney, liver, lung, AIDS and cancer), active infection, schizophrenia, and other psychiatric disorders, and seizure, which were detected by 2 psychiatrists.

**Figure 1:**
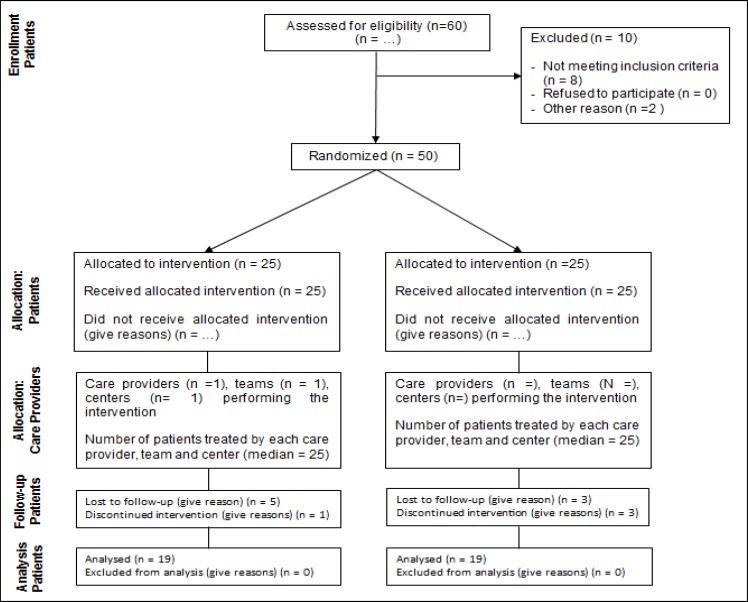
Flow Diagram of the Study

Using permuted blocked randomized clinical trial, patients were divided into equal groups of placebo and probiotics with single-blinding in randomization. Allocation concealment was based on block size of 4. Patients were considered in blocks of 4 in their admissions in the hospital according to way A B A B (A: placebo, B: probiotic) in randomization. Patients in both groups were allowed to receive lithium oxide, with a maximum dose of 900 mg per day, sodium valproate, with a maximum dose of 1200 mg per day, and, if necessary, risperidone. Probiotic group received a probiotic capsule (containing 1.8×109 CFU/capsule Bifidobacterium bifidum, Bifidobacterrium lactis, Bifidobacterium langum, and Lactobacillus acidophilus), and placebo group received a placebo capsule of the same properties as probiotics in terms of color, size, and weight (containing maltodextrin). Patients consumed the capsules daily and continued taking them after discharge. Drugs were kept in the refrigerator at the hospital and at home. Probiotics and placebo capsules were prepared by Tak-Gen-Zist (Tehran, Iran). Duration of intervention was 8 weeks (18), and the questionnaires were completed by a psychiatry resident at the beginning of the study, before the intervention, at week 4, and week 8. Figure 1 displays the consort flow diagram of the study.

Study instrument: Mania Rating Scale (YMRS)-Persian version and Hamilton's Depression Rating Scale-Persian version with 17 items (HDRS-17) were used in this study.

***Young Mania Rating Scale (YMRS)***

This 11-item scale was developed by Young et al and is scored based on the Likert scale from zero (normal) to 4 (abnormal). (14). In the sample of Iranian patients, its concurrent validity was obtained by correlation with Composite International Diagnostic Interview (CIDI) (0.87) and Cronbach's alpha coefficient of 0.72 and 0.63 for patient and healthy groups, respectively (15).

***Hamilton's Depression Rating Scale (HDRS)***

This scale was developed by Hamilton to measure the severity of depression symptoms in a clinical interview. The scale has 24 items and is graded by a clinician based on the Likert scale. Its susceptibility to exacerbate depression cases was found to be 87%, and its sensitivity and specificity were found to be 62.4% and 92%, respectively (16). In Iran, the validity of this tool has been reported through correlation with Beck’s Depression Scale and Ineffective Attitudes Scale of 0.55 and 0.39, respectively, and the reliability among the evaluators was 0.95 (17).

***Ethical Consideration***

All procedures of this study were based on the Helsinki Declaration. This study was approved by the Ethics Committee of Kerman University of Medical Sciences, with the ethical license number IR.KMU.AH.REC.1397.009, and at the same time it was registered in the Register of Clinical Trials, with registration number IRCT20180819040836N1. The intervention was explained to patients or their legal guardians and informed consent was obtained. 

***Data Analysis***

Data were analyzed using SPSS version 20 software (IBM Corp., Armonk, NY, USA). The results were analyzed by independent t test, Mann-Whitney test, and Repeated Measure ANOVA test. P < 0.05 was considered statistically significant.

## Results

In this study, 50 patients with bipolar disorder were hospitalized and selected. They were divided into 2 groups: probiotic (n = 25) and placebo (n = 25). During the first 4 weeks of the study, with a decrease of 6 in each group because of family consent to patient’s discharge despite doctor's advice and discontinuing the medication, the final sample in each group was reduced to 19. 

The mean age of the probiotic group was 38.9 years, with the standard deviation of 9.83, and that of the placebo group was 35 years, with the standard deviation of 8.18. There were no significant differences between the 2 groups in age and gender, and p value was 0.19 and 0.7, respectively.

**Diagram 1:**
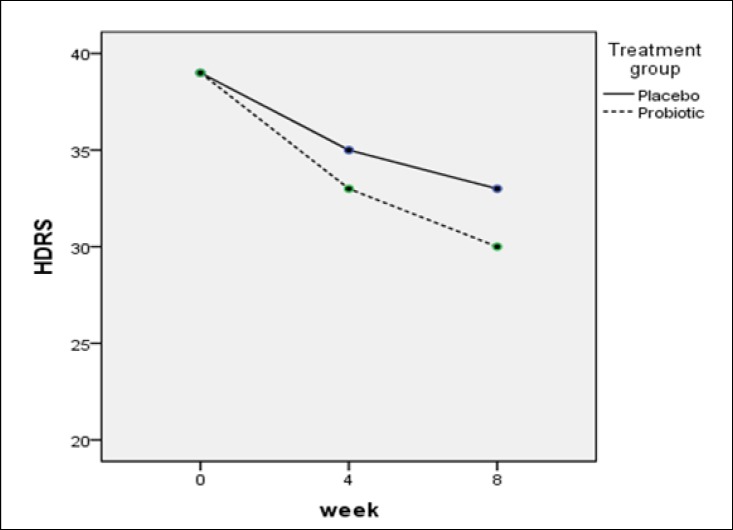
Changes in Hamilton's Depression in Both Groups

**Diagram 2:**
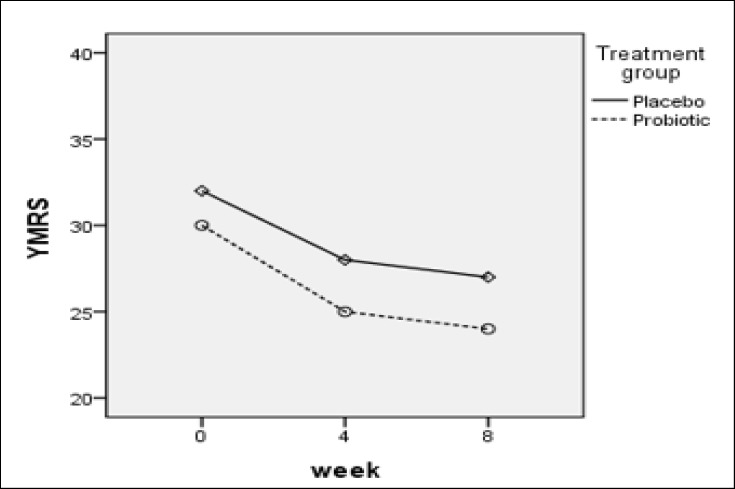
Changes in Yang Mania Results in the 2 Groups

According to Table 1, probiotic consumption significantly decreased the scores of Young Mania (p value = 0.001) and Hamilton (p value = 0.001) questionnaire over time in bipolar type 1 recipient group receiving probiotics. Similarly, in the placebo group, the Young Mania (p value = 0.002) and Hamilton (p value = 0.004) questionnaire scores decreased over time in type 1 bipolar disorder group, and this difference was higher in the probiotic group than in the placebo group (diagram 1 & 2). 

There was no significant difference in the mania (P value = 0.2) and depression (P value = 0.5) scores between placebo and probiotics patients with type 1 bipolar disorder.

## Discussion

In this randomized placebo-controlled study, the effect of taking probiotic supplements was evaluated on 38 patients with type 1 bipolar disorder. In this study, using the Hamilton’s depression and Young’s Mania Questionnaires at the beginning of the study (zero or baseline), 4 and 8 weeks after the onset of the intervention, the effects of probiotic consumption were evaluated on the severity of mania and depression in bipolar patients. The results of this study indicated that probiotic consumption did not significantly change the severity of depression and mania between the 2 study groups, meanwhile probiotic consumption significantly reduced the severity of depression and mania over time in the probiotic group.

Inconsistent with this research, in the study of Akkasheh et al on 40 patients with MDD taking daily probiotics (Bifidobacterium bifidum, Lactobacillus casei, Lactobacillus acidophilus) for 8 weeks, significant reduction was found in depression symptoms (18). The Reininghaus et al study in 2018 titled " The Impact of Probiotic Supplements on Cognitive Parameters in Euthymic Individuals With Bipolar Disorder: A Pilot Study " was conducted in Australia on 20 patients with bipolar disorder, Bifidobacterium bifidum, and lactobacillus. Using the Young Mania Scale, it was found that psychomotor processing speed and attention significantly improved and significant reductions in mania symptoms were achieved 1 and 3 months after the commencement of the study (19). Likewise, in the present study, there was a decrease in the Young Mania Rating Scale over time.

The inconsistency of the results of this study with those of other studies may be due to the following reasons: the difference in sample size, bacterium’s type and strain, duration of study, the severity of the disease, and the simultaneous use of mood stabilizing drugs, dissimilarities in patient evaluation methods, geographical location of patients, and genetics. About the relationship between the strains of the probiotics and their efficacy on other diseases or disorders some studies were found. For instance, as some studies have suggested that only some probiotic strains have beneficial effects on IBS, diarrhea associated with antibiotics and H. pylori (13) infection and neonatal colic (14), and only some bifidobacteria for treatment of colitis ulcer are promising (15).

Probiotic supplementation as an adjuvant treatment in some mental disorders (26, 27) is associated with a lower rate of rehospitalization in patients who have been recently discharged following hospitalization for mania, receiving 24 weeks of adjunctive probiotics (Lactobacillus rhamnosus strain GG and Bifidobacterium animalis subsp. lactis strain Bb12) (20), emphasizing longer duration of treatment. Overall, decrease in scores of Young Mania and Hamilton Questionnaire over time in bipolar type 1 confirmed that targeting the microbiome may be an effective treatment paradigm for bipolar disorder (21) in parallel with the investigations that suggest bipolar disorders are associated with reduced microbial diversity and show global community differences compared to nonpsychiatric comparison samples (22).

Global gut microbial community differences in BD showed decreased fractional representation of Faecalibacterium and an unclassified member from the Ruminococcaceae family, both from the phylum Firmicutes (21), while these probiotic microbiomes were not consumed by the patients in this study.

**Table 1:** Comparison of Changes in YMRS and Hamilton HDRS Scores in the 2 Groups

		**M(SD)** **‎**			**P**	**p**
**Variables**	**Drug ** **Group**	**First Day**	**Four Weeks After ** **Intervention**	**Eight Weeks After ** **Intervention**	**Within ** **Group**	**Between ** **Group**
YMRS	Probiotics	30.2(5.8)	25.7(5.6)	24.3(6.1)	<0.001	0.2
	placebo	32.8(7.2)	28.6(7.1)	27.0(7.5)	P=0.002	
HDRS	Probiotics	‎39.0(8.5)‎	33.1(8.9)	30.4(9.6)	‎<0.001‎	0.5
	placebo	39.3(10.8)	35.6(9.8)	33.5(9.7)	P=0.004	

## Limitation

In this study, small sample size, the duration and the dose of probiotics consumed, and the possibility of interference between mood stabilizing drugs and the effects of probiotics could be cited as limitations.

In addition, a number of patients did not continue consuming probiotics after discharge; therefore, the researchers had to select new patients from the community’s psychiatric center based on the study’s inclusion and exclusion criteria.

## Conclusion

According to the results of this study and based on the Hamilton’s Depression and Young Mania Scale, the use of probiotics mentioned in this study had no significant effect on improvement and treatment of patients group compared to placebo group. On the other hand, consumption of probiotics did not have a negative effect on patients and was completely tolerable for patients. Therefore, to better evaluate probiotics’ effects as a complementary treatment to decrease bipolar disorder severity, future studies should be conducted with different strains and increase the dose and duration of probiotic consumption. It is also recommended to evaluate cognitive functions and quality of life of patients and longevity in treatment (recurrence prevention).
